# Supplementary material for: Impact of e-money on money supply: Estimation and policy implication for Bangladesh
Source: PLoS One. 2022 Apr 26;17(4):e0267595. doi: 10.1371/journal.pone.0267595 (PMC9041864; doi:10.1371/journal.pone.0267595)
Supplement: Attachment — Submitted filename: Response-To-Reviewers.docx [file pone.0267595.s001.docx]

**Reviewer’s comment #1:**

The abstract should reserve more space to the quantitative results (e.g. the ~10% of money supply due to MFS), and a bit less space to the qualitative argument against capping interest rates, to reflect the importance of the results and the space reserved in the manuscript to both.

**Answer:**

We have rewritten the abstract according to the reviewer’s suggestion. It now reads as follows:

With the rapid proliferation of mobile telephony and the establishment of an IT-enabled payment and settlement system, Bangladesh nowadays is experiencing a remarkable growth in the usage of mobile financial services (MFS). As more and more people are opting to use this service, a huge number of mobile accounts are opened every day and a substantial amount of money is deposited, withdrawn and transferred frequently through the mobile network. This ever-increasing amount of mobile money flowing through the network may have a sizeable impact on the overall money supply of the country. Thus far, no systematic study has been conducted to quantify the impact of the mobile money on the conventional money supply of Bangladesh. In this study, we attempt to quantify the contribution of mobile money on the money supply which is an important quantity-based nominal anchor of monetary policy in Bangladesh. Apart from deriving algebraic relationships between money supply and e-money, here we have empirically shown that during the 03 years span of 2018-2021, MFS transactions account for nearly 10.88% and 11.29% of total narrow and broad money supply of Bangladesh as on January 2021. Besides, we also qualitatively discuss the impact of e-money on an important price-based nominal anchor of monetary policy in Bangladesh, i.e., interest rate. Based upon the above discussion, here we argue that MFS can act as an effective tool to slash interest rate by a reasonable proportion through adding significantly to the overall supply of money in Bangladesh.

**Reviewer’s comment #2:**

Some space could be dedicated in the discussion to limitations or possible extension of the work. In particular, a natural extension of this study, to better understand the dynamics and importance of MFS transactions, could be to study the network of these MFS transactions, to better understand the role played by different kinds of agents (merchants, people ecc) and the effective inclusivity of such system (how many rural people are actually active, what's the distribution of total money in accounts etc.).

**Answer:**

We have added a separate section titled ‘Future Work’ which reads as follows:

Current study can be reasonably extended to a number of different directions. For example, although we have substantiated the money creation process through MFS transactions in the context of Bangladesh, we did not analyze the relative inclusivity of such a system, i.e., whether both the urban and rural population participate equally in the money creation process or it is the hegemony of the urban population only. Besides, the role played by different economic agents, e.g., regulators, MFS providers, merchants, agent points, end users etcetera in the process and the network dynamics of the whole ecosystem are yet to be investigated. Moreover, although we have quantified the impact of MFS transactions on the overall money supply, the responsiveness of different monetary aggregates to changes in mobile money is left unattended for the purpose of current study. A more econometric paper may resort to analyze the responsiveness of narrow money (M1), broad money (M2) and other aggregates alike to changes in e-money possibly under a structural VAR framework which is beyond the scope of the current study.

**Journal Requirement #1:**

Please ensure that your manuscript meets PLOS ONE's style requirements, including those for file naming. The PLOS ONE style templates can be found at 
https://journals.plos.org/plosone/s/file?id=wjVg/PLOSOne_formatting_sample_main_body.pdf and 
<https://journals.plos.org/plosone/s/file?id=ba62/PLOSOne_formatting_sample_title_authors_affiliations.pdf>

**Answer:**

We have reformatted our manuscript by meticulously following PLOS ONE’s style requirement. Precisely, we have reformatted it using PLOS ONE’s latex template provided in the journal website.

**Journal Requirement #2:**

We suggest you thoroughly copyedit your manuscript for language usage, spelling, and grammar. If you do not know anyone who can help you do this, you may wish to consider employing a professional scientific editing service.

Whilst you may use any professional scientific editing service of your choice, PLOS has partnered with both American Journal Experts (AJE) and Editage to provide discounted services to PLOS authors. Both organizations have experience helping authors meet PLOS guidelines and can provide language editing, translation, manuscript formatting, and figure formatting to ensure your manuscript meets our submission guidelines. To take advantage of our partnership with AJE, visit the AJE website (http://aje.com/go/plos) for a 15% discount off AJE services. To take advantage of our partnership with Editage, visit the Editage website (www.editage.com) and enter referral code PLOSEDIT for a 15% discount off Editage services.  If the PLOS editorial team finds any language issues in text that either AJE or Editage has edited, the service provider will re-edit the text for free.

Upon resubmission, please provide the following:
 
The name of the colleague or the details of the professional service that edited your manuscript

A copy of your manuscript showing your changes by either highlighting them or using track changes (uploaded as a *supporting information* file)

A clean copy of the edited manuscript (uploaded as the new *manuscript* file)

**Answer:**

1. We took help from our colleague Dr. Md Nasim Adnan to copyedit the entire manuscript.
2. We have uploaded our manuscript highlighting all the changes that have been made. It is uploaded as a *supporting information* file.
3. A clean copy of the edited manuscript titled *manuscript* is also uploaded into the submission site.
4. A separate copy of the manuscript with track changes for review purpose has been uploaded as *Revised Manuscript with Track Changes*.

**Journal Requirement #3:**

Please review your reference list to ensure that it is complete and correct. If you have cited papers that have been retracted, please include the rationale for doing so in the manuscript text, or remove these references and replace them with relevant current references. Any changes to the reference list should be mentioned in the rebuttal letter that accompanies your revised manuscript. If you need to cite a retracted article, indicate the article’s retracted status in the References list and also include a citation and full reference for the retraction notice.

**Answer:**

Most of the web links in the bibliography section has gone outdated and are replaced with their new replacement links. This is due to the fact that two of the websites that are cited frequently in the current piece, namely, Bangladesh Bank (BB) and Bangladesh Telecommunication Regulatory Commission (BTRC) have updated their websites very recently rendering majority of the previously cited links invalid. We have replaced every old hyper-link previously cited in this article by its new URL. Moreover, we have re-written the bibliographic section of the article following Harvard style of referencing as suggested by the journal. All the in-text citations have also been modified to reflect Harvard style of referencing.
